# Reduced Gut Acidity Induces an Obese-Like Phenotype in *Drosophila melanogaster* and in Mice

**DOI:** 10.1371/journal.pone.0139722

**Published:** 2015-10-05

**Authors:** Wei-Sheng Lin, Cheng-Wen Huang, You-Sheng Song, Jui-Hung Yen, Ping-Chang Kuo, Sheng-Rong Yeh, Hung-Yu Lin, Tsai-Feng Fu, Ming-Shiang Wu, Horng-Dar Wang, Pei-Yu Wang

**Affiliations:** 1 Graduate Institute of Brain and Mind Sciences, College of Medicine, National Taiwan University, Taipei, Taiwan; 2 Institute of Neuroscience, National Chengchi University, Taipei, Taiwan; 3 Department of Microbiology and Immunology, Indiana University School of Medicine, Fort Wayne, Indiana, United States of America; 4 Department of Applied Chemistry, National Chinan University, Nantou, Taiwan; 5 Department of Internal Medicine, National Taiwan University Hospital, National Taiwan University, Taipei, Taiwan; 6 Department of Pediatrics, National Taiwan University Hospital Yun-Lin branch, Yun-Lin, Taiwan; 7 Institute of Biotechnology, National Tsing Hua University, HsinChu, Taiwan; 8 Institute of Systems Neuroscience, National Tsing Hua University, HsinChu, Taiwan; 9 Department of Life Science, National Tsing Hua University, HsinChu, Taiwan; National Health Research Institutes, TAIWAN

## Abstract

In order to identify genes involved in stress and metabolic regulation, we carried out a *Drosophila* P-element-mediated mutagenesis screen for starvation resistance. We isolated a mutant, *m2*, that showed a 23% increase in survival time under starvation conditions. The P-element insertion was mapped to the region upstream of the *vha16-1* gene, which encodes the c subunit of the vacuolar-type H^+^-ATPase. We found that *vha16-1* is highly expressed in the fly midgut, and that *m2* mutant flies are hypomorphic for *vha16-1* and also exhibit reduced midgut acidity. This deficit is likely to induce altered metabolism and contribute to accelerated aging, since *vha16-1* mutant flies are short-lived and display increases in body weight and lipid accumulation. Similar phenotypes were also induced by pharmacological treatment, through feeding normal flies and mice with a carbonic anhydrase inhibitor (acetazolamide) or proton pump inhibitor (PPI, lansoprazole) to suppress gut acid production. Our study may thus provide a useful model for investigating chronic acid suppression in patients.

## Introduction

Aging and metabolic syndrome are among the major issues in contemporary medicine. Although the mechanisms underlying these health problems remain incompletely understood, it has been demonstrated that aging and metabolism are intimately related, and that gut homeostasis plays an important role in regulation of these processes [1–3]. Recent studies have suggested that chronic (at least 10 months), but not short term (4 months), PPI treatment is associated with undesirable weight gain [4, 5]. Similarly, a suboptimal weight loss after gastric bypass bariatric surgery was found in two separate cohorts of PPI users [6]. Acid homeostasis in the gut may thus play a critical role in metabolic regulation.

Gastric acid is produced by the parietal cells of the stomach, and not only facilitates digestion and absorption of nutrients, but also helps to prevent bacterial overgrowth and enteric infection. In the *Drosophila* digestive tract, the midgut region is considered to be the equivalent of the mammalian stomach/small intestine. Specialized acid-producing cells, the copper cells of the midgut, were originally identified by their orange florescence in copper-fed larvae and their ability to accumulate radioactively-labeled cooper [7]. Copper cells show several striking similarities to mammalian gastric parietal cells in morphological studies, and they also co-localize with the acidic region of *Drosophila* midgut [7, 8]. Moreover, acid secretion is not detectable in mutant flies with perturbed copper cell differentiation [9]. Copper cells of the fly midgut thus appear to be structurally and functionally related to mammalian parietal cells.

Unlike parietal cells of the mammalian stomach that rely on H^+^K^+^-ATPase (of the P-type H^+^ ATPase family, *pha*) for acid secretion, *Drosophila* copper cells use V-type H^+^ ATPase (*vha*) for acid secretion [10]. No *pha* has thus far been shown to be expressed in *Drosophila*. Vacuolar-type H^+^ ATPases are multisubunit proton pumps comprising two functional parts, an ATP catalytic V_1_ complex (subunits A to H), and a proton-translocating V_0_ complex (subunits a, c, d and e). *vha* is an ATP-hydrolyzing enzyme that can transfer energy to proton gradients across diverse biological membranes, thus permitting regulation of the acidity of an organelle or of the extracellular side of the membrane. Although it has been suggested that gut acidification in insects may work through *vha* [11], it remains to be shown whether *vha* is actively involved in acid production in the *Drosophila* midgut. Here, we report that *vha16-1* is a critical component of acid production in the fly midgut. Genetic and pharmacological approaches for acid suppression in the fly and in mice both induced increased body weight and lipid accumulation, and in flies, were also accompanied by accelerated aging. Our results may have critical implications for the use of chronic acid suppression in a clinical setting.

## Materials and Methods

### Flies and life span assays

*w* ^*1118*^, *EP2372*, *P[Δ2–3]*, *P112087*, *Cyo/sp;TM3*,*Ser/TM6*, *Cop-Gal4 (NP3270)*, *daughterless-Gal4* and *UAS-GFP* fly stocks were obtained from the Bloomington *Drosophila* Stock Center. *UAS-vha16-1* was generated from a full-length *vha16-1* cDNA and subcloned into the pUAST vector as previously described [12]. All flies were raised on standard sucrose/yeast/cornmeal food and were backcrossed into the *w* ^*1118*^ background for at least 5 generations, as described previously [13]. For the life span assays, flies that had eclosed within 48 hours (approximately 100 males and 100 females) were transferred to a 1-liter population cage and maintained in a humidified, temperature-controlled incubator with 12-hour on/off light cycle at 25°C [14]. Fresh food was provided every other day, and the number and sex of dead flies were scored. Fly food contained 5% dextrose, 5% yeast, 2% agar, and 0.23% Tegosept (Apex). 500uM lansoprazole (Takeda Pharmaceuticals Taiwan), acetazolamide (Sigma) or vehicle control was added to the foods described in the experiment.

### Genetic screen for starvation resistance

The P-element-containing line *P112087* was crossed to a constitutively expressed transposase source *P[Δ2–3]* in order to excise and transpose the P-element to other chromosomal locations in progeny. The transposase was then removed by crossing F1 progeny to a balancer line *Cyo/sp;TM3*,*Ser/TM6*. Individual flies having the P-element integrated on the 2^nd^ or 3^rd^ chromosome were established as stock lines. For starvation challenge, ten-day-old flies that had been maintained on regular food were transferred to vials containing 2% agar and the number of dead flies was counted every 3 to 4 hours.

### Inverse PCR and mRNA quantification

To map the insertion site of the P-element, genomic DNA was isolated from more than 30 flies using WelPrep DNA isolation kit (Welgene). DNA was then digested with DpnII (NEB) and ligated with T4 DNA ligase (Fermentas). PGaw2 5’-CAGATAGATTGGCTTCAGTGGAGAC–3’, PGaw3 5’-CGCATGCTTGTTCGATAGAAGAC–3’, Plw3 5’-TAACCCTTAGCATGTCCGTGGGGTTTG–3’, and PRY4 5’-CAATCATATCGCTGTCTCACTCA -3’ primers were used to amplify the flanking regions of the P-element. A PCR product of approximately 2000 base pair was isolated and sequenced. The P-element was mapped to nucleotide 6632424 on the 2^nd^ chromosome of *Drosophila melanogaster*. The P-element insertion was also verified by specific primers GT-F 5’-CCGCACGTAAGGGTTAATGTTTT–3’, GT-R 5’-TTTTGCACGGATGAATCTGAATG–3’, *PGawB*-F 5’-GTGATTGGTTTTGGGTGGGTAA -3’ and *PGawB*-R 5’-TGTTCAGATGCTCGGCAGATG–3’.

For mRNA quantification, total RNA was prepared from at least 30 flies using the NucleoSpin RNA Kit (Macherey-Nagel), and the RNA was converted to cDNA as described previously [15]. Quantitative polymerase chain reaction (qPCR) was carried out using a StepOnePlus Real-Time PCR System (Applied Biosystems), SYBR Green Master Mix (Fermentas), and gene-specific primers 5’-GGAACTCACGAAGCAAGTGTTGA–3’ and 5’-AAAACCGCACCATTGGATACAT–3’ for *vha16-1*, and 5’-AATGGGTGTCGCTGAAGAAGTC–3’ and 5’-GACGAAATCAAGGCTAAGGTCG–3’ for glyceraldehyde 3-phosphate dehydrogenase (GAPDH). A two-step PCR reaction was carried out with denaturation at 95°C for 15 seconds, annealing and extension combined at 60°C for 1 minute, in a total of 40 cycles. The mRNA expression level of each target gene compared to GAPDH was quantified by subtraction: Ct (specific gene)—Ct (GAPDH) = ΔCt. A difference of one PCR cycle equates to a two-fold change in mRNA expression level. The uniqueness of amplicons was confirmed using dissociation curves.

### Gut acidity, total triglycerides, body weight, feeding and fecundity measurements in flies

To measure fly gut acidity, 10-day-old flies were fed with yeast paste containing 1% bromophenol blue for 24 hours, and midgut acidity was observed under a dissecting microscope. To measure total triglycerides, ten 10-day-old flies were homogenized in PBS containing 0.05% Tween 20 and centrifuged at 16,000 g. Total triglycerides were measured using a triglyceride test kit (Fortress Diagnostics). For body weight measurements, 10-day-old flies were anesthetized by CO_2_ and weighed immediately, using a microbalance (Sartorius). Female fecundity was determined by daily counting of eggs produced by 3 mating pairs. Flies were passed daily to new vials containing appropriate food, and the number of eggs laid was counted and recorded for the first 14 days of adult life. For the feeding assays, 10-day-old flies were transferred to fresh vials with regular food containing 0.5% FD&C no. 1 blue food dye. After 6 hours, 10 flies were homogenized in a single tube containing PBS, and the amount of ingested dye was determined by spectrophotometer for dye absorbance at 620nm.

### Mouse experiments

All experimental protocols followed the local animal ethics regulations and approved by National Taiwan University College of Medicine and College of Public Health Institutional Animal Care and Use Committee (IACUC). C57BL/6 male mice were obtained from National Taiwan University College of Medicine Laboratory Animal Center and maintained in an animal room with controlled temperature at 22–24°C and humidity at 50–55% under 12 hr light/dark cycle. All mice were fed *ad libitum* with standard pelleted mice chow (LabDiet 5058, PicoLab). For chronic acid suppression, mice received a daily subcutaneous injection of lansoprazole for 3 to 4 months. Food intake, water consumption and change in body weight were monitored regularly. Blood from tails of mice was collected at different time points and serum total triglycerides and cholesterol were measured using commercial kits (Fortress Diagnostics). For stomach acidity measurements, the mouse stomach was removed and washed with 1ml PBS. Gastric contents were collected and pH of the collected gastric juice was measured using a pH meter (Sartorius).

### Statistics

All data are expressed as mean ± SEM. Survival curves were analyzed by the Kaplan-Meier procedure and log-rank test. Data for all other assays were analyzed using one-way ANOVA or Student’s t test.

## Results

### Isolation of *vha16-1* mutant flies

**Fig 1:**
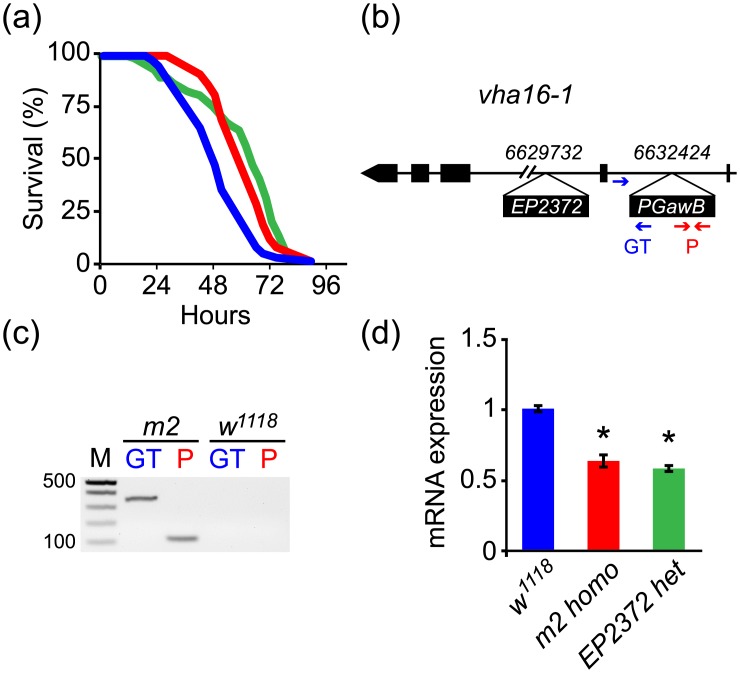
*vha16-1* mutation induces starvation resistance in flies. (a) Enhanced survival of *m2* homozygous (red) and *EP2372* heterozygous (green) mutant flies on starvation challenge compared to control flies (*w* ^*1118*^, blue). Statistical analysis is shown in Table 1. (b) Schematic diagram of *vha16-1* gene illustrating the insertion site of P-elements. (c) The *P[GawB]* insertion in *m2* mutant flies was further verified by specific primer sets. The GT primer set detected a PCR product spanning the *vha16-1* gene and *P[GawB]* fragment, and P generated a PCR product within the *P[GawB]*. (d) *vha16-1* mRNA is significantly down regulated in *m2* homozygous mutant flies (red) and *EP2372* heterozygous (green) mutant flies compared to control flies (*w* ^*1118*^, blue). Experiments were done in triplicate and each replicate contained more than 30 flies for each group. *, *P* < 0.05, compared to the control group.

To identify novel genes involved in metabolic regulation, we set up a genetic screen for starvation resistance using P-element-mediated mutagenesis in *D*. *melanogaster*. A total of 696 mutant lines were generated, and a mutant, dubbed *m2*, was found to have prolonged survival under food-deprived conditions, and was homozygous viable and fertile (Fig 1a and Table 1). Inverse PCR followed by sequencing showed that the landing site of the P-element for this mutant is in the first intron of the *vha16-1* gene (Fig 1b), which encodes the c subunit of *vha*. The insertion site of *P[GawB]* was further confirmed by PCR, which detected an approximately 350 base pair PCR product spanning the *vha16-1* gene and a *P[GawB]* fragment (Fig 1b and 1c), and an approximately 120 base pair PCR product within the *P[GawB]*. This P-element insertion produces a hypomorphic mutation of the *vha16-1* gene, since we detected approximately 40% down-regulation of *vha16-1* mRNA in *m2* homozygous mutant flies (Fig 1d).

**Table 1:** The effects of acid suppression on starvation resistance and life span (LS) in *Drosophila melanogaster*.

	Strain	Number	Mean LS (day/hr)	Extension (%)
Starvation				
	*w* ^*1118*^	200	50.9 hr	
	*m2*	200	62.6 hr	22.3*
	*EP2372*	199	60.7 hr	19.3*
Life span				
	*w* ^*1118*^	314	48.3 day	
	*m2*	327	39.0 day	-19.3*
	*EP2372*	321	43.5 day	-9.9*
	*w* ^*1118*^	217	50.5 day	
	*lansoprazole*	181	38.5 day	-23.8*

* *P* value<0.01 compared to genetic matched control or vehicle control by log-rank test.

For comparison, an additional *vha16-1* mutant *EP2372* was obtained from the Bloomington Drosophila stock center. *EP2372* is homozygous lethal, but heterozygous mutant flies showed reduced *vha16-1* mRNA expression and increased starvation resistance, similar to our results for *m2* homozygous mutant flies (Fig 1a, 1b and 1c).

### *vha16-1* mutation reduces gut acidity in flies

**Fig 2:**
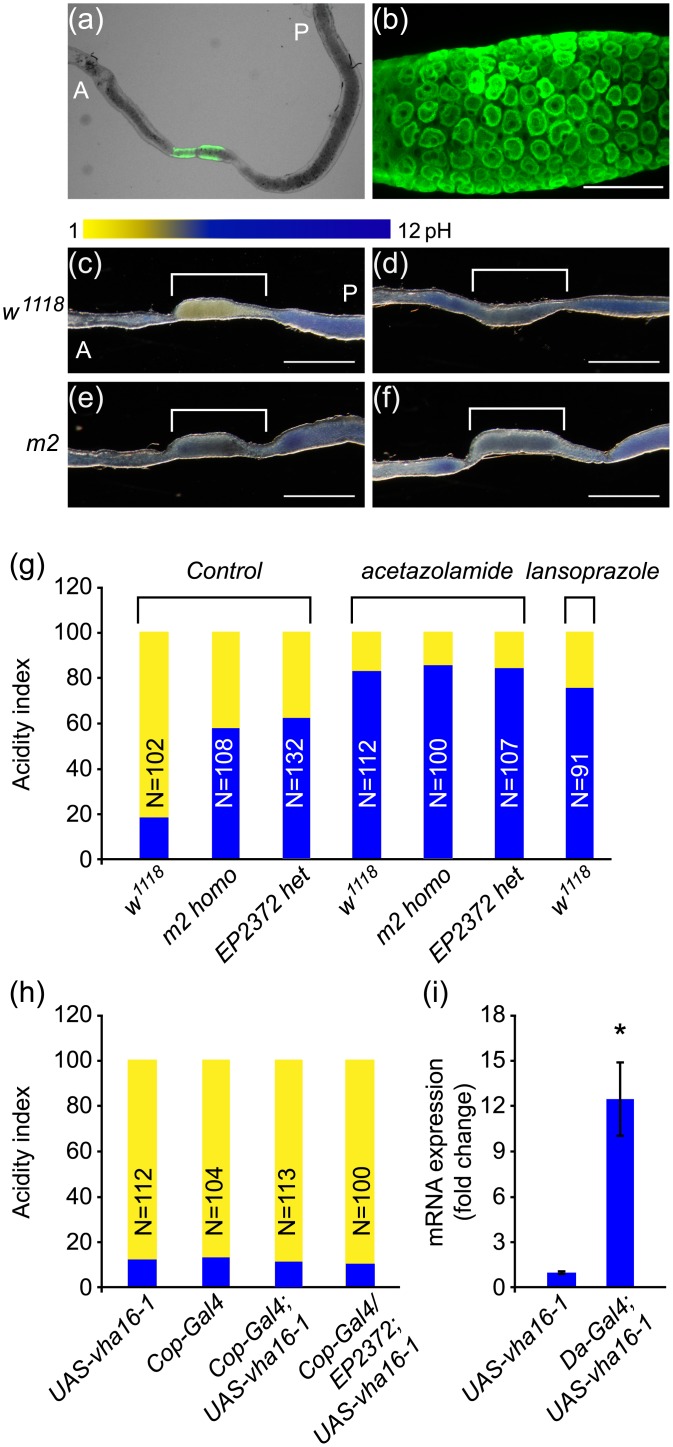
*vha16-1* mutation reduces gut acidity in flies. (a and b) *m2>UAS-GFP* reporter flies showing that *vha16-1* was highly expressed in the *Drosophila* midgut region. Scale bars = 100 μm. (c-f) Representative images showing that *m2* homozygous mutant flies (e) displayed reduced midgut acidity compared to control flies (c, *w* ^*1118*^). The midgut acidity can also be diminished by feeding both control flies (d) and *m2* homozygous mutant flies (f) with carbonic anhydrase inhibitor (acetazolamide). Scale bars = 0.5 mm. The anterior and posterior regions of *Drosophila* midgut are marked as “A” and “P”, respectively. (g) Quantitative results showing that majority of *m2* homozygous, *EP2372* heterozygous mutant flies, and acetazolamide- or lansoprazole-treated flies had diminished midgut acidity compared to control flies. (h) *vha16-1* overexpression specifically in copper cells (*Cop-Gal4/;UAS-vha16-1*) did not affect the midgut acidity of flies, but did recover the midgut acidity of *EP2372* heterozygous mutant flies to normal level. (i) Effectiveness of the *UAS-vha16-1* constructs used for *vha16-1* overexpression was verified by crossing *daughterless (Da)-Gal4* with *UAS-vha16-1* flies. *vha16-1* mRNA significantly increased in *Da-Gal4;UAS-vha16-1* flies compared to control (*UAS-vha16-1*) flies. Experiments were done in triplicate and each replicate contained more than 30 flies for each group. *, *P* < 0.05, compared to the control group.

Since *m2* mutant flies carrying *P[GawB]* constitutively express the yeast transcription activator protein Gal4, we crossed them to mutant flies carrying a UAS-GFP construct. The progeny reporter flies displayed a high level of GFP expression in a circumscribed segment of the central midgut in adult flies (Fig 2a and 2b). This segment corresponds to the copper cell region, where *vha* is highly expressed in the apical plasma membrane, and is responsible for acidification of the midgut lumen [16, 17]. To evaluate whether midgut acidity is affected in *vha16-1* mutants, flies were fed bromophenol blue (BPB, a pH indicator) for 24 hours. BPB changes color from yellow at pH = 3.0 to blue at pH = 4.6. We found that 56.06% of *m2* homozygous mutant flies and 60.61% of *EP2372* heterozygous mutant flies showed diminished midgut acidity, compared to 19.23% of control *w* ^*1118*^ flies (Fig 2c–2g). Although *vha16-1* overexpression specifically in copper cells did not affect midgut acidity of flies, it did effect recovery of midgut acidity of *EP2372* heterozygous mutant flies to the normal level (Fig 2h). Moreover, a majority of *w* ^*1118*^ control flies and *vha16-1* mutant flies fed with acetazolamide or lansoprazole also exhibited diminished midgut acidity (Fig 2g). Lansoprazole is has been shown to inhibit gastric and vesicular acidity through inhibition of both *vha* and *pha* [18, 19].

### Gut acid suppression increases lipid accumulation and body weight in flies

**Fig 3:**
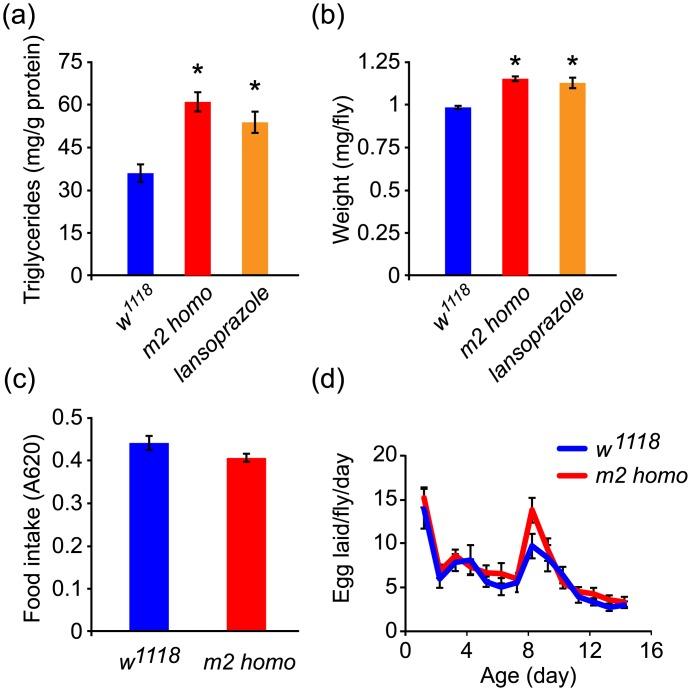
Midgut acid suppression induces an obese-like phenotype in flies. (a and b) *m2* homozygous mutant flies (red) and lansoprazole-treated flies (orange) show increased triglyceride accumulation (a) and body weight (b) compared to control flies (*w* ^*1118*^, blue). (c and d) Food intake and female fecundity are not altered in *m2* homozygous mutant flies (red) compared to control flies (*w* ^*1118*^, blue). Data were collected from 8–10 replicates for each group. Each replicate contained 10 flies for triglyceride (a), body weight (b) and feeding assays (c), and 3 mated pairs for female fecundity measurements (d). *, *P* < 0.05, compared to the control group.

Starvation resistance in animals is generally associated with altered metabolism. For instance, animals having increased lipid accumulation or body weight are often resistant to food deprivation because of their greater nutrient storage [13, 20]. We examined triglycerides and body weight in flies as a measure of nutrient storage and ability to resist starvation. We found that *m2* homozygous mutant flies have an elevated level of triglycerides and increased body weight compared to control *w* ^*1118*^ flies (Fig 3a and 3b). Intriguingly, chronic acid suppression by lansoprazole also induced these phenotypes, similar to what is seen in *m2* homozygous mutant flies (Fig 3a and 3b).

Increased nutrient storage upon gut acid suppression was not associated with alterations in feeding behavior, since the feeding rate for *m2* homozygous mutant flies was comparable to that of control *w* ^*1118*^ flies (Fig 3c). Although female fecundity is also linked to energy availability in animals, this is not a factor in *m2* homozygous mutant flies, as normal egg production was observed in the first 2 weeks of female adult life (Fig 3d).

### Gut acid suppression reduces life span in flies

**Fig 4:**
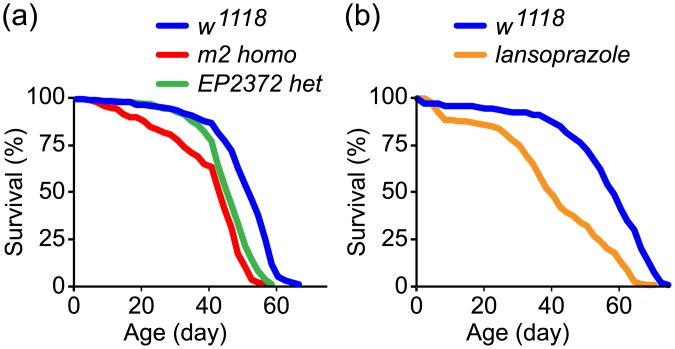
Midgut acid suppression shortens life span in flies. (a) Shortened life spans in *m2* homozygous mutant flies (red) and *EP2372* heterozygous mutant flies (green) compared to control flies (*w* ^*1118*^, blue). (b) Shortened life spans in lansoprazole-treated flies (orange) compared to control flies (*w* ^*1118*^, blue). Statistical analysis is shown in Table 1.

Because metabolism is considered to be a central component of life span regulation, it is therefore crucial to determine how life span is affected in flies that have decreased midgut acidity. We found that median and maximal life span were significantly decreased in both *m2* and *EP2372* mutants, as well as in lansoprazole-treated flies, compared to control *w* ^*1118*^ flies (Fig 4a and 4b and Table 1).

### Gut acid suppression induced an obese-like phenotype in mice

**Fig 5:**
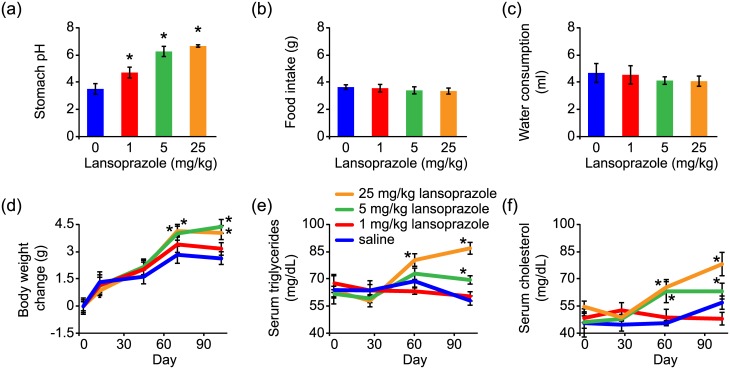
Gastric acid suppression induces an obese-like phenotype in mice. (a) Increased stomach pH in mice receiving lansoprazole treatment. (b and c) Chronic lansoprazole treatments do not affect food intake (b) or water consumption (c) of mice. (d-f) Chronic lansoprazole treatments gradually induce increased body weight, and elevated levels of serum triglycerides and cholesterol. Data were collected from 7 mice for each group. *, *P* < 0.05, compared to the saline group.

We further verified whether acid suppression in mice could induce a similar phenotype to that observed in flies. We treated mice with lansoprazole for 3 to 4 months, and found that stomach acidity was significantly reduced in a dosage-dependent manner (Fig 5a). Over the course of lansoprazole treatment, we found that serum triglycerides and cholesterol gradually increased for mice on higher doses of lansoprazole (5 and 25 mg/kg) but not on the lower dose of lansoprazole (1 mg/kg). Levels of serum triglycerides and cholesterol were significantly higher 60 days after receiving lansoprazole (Fig 5e and 5f). Moreover, body weight was also increased in mice treated with higher doses of lansoprazole (Fig 5d). Neither food intake nor water consumption was altered in mice treated with lansoprazole (Fig 5b and 5c).

## Discussion

Our study demonstrates that flies and mice consistently exhibit an obese-like phenotype, that is accompanied by accelerated aging in flies, when gut acid production is suppressed by either genetic or pharmacologic means. On the other hand, the resulting elevated store of triglycerides probably confers starvation resistance upon these flies [21, 22]. The relationships among fat storage, starvation resistance, and life span are complex and sometimes counterintuitive. Obesity and increased stores of triglycerides can be associated with either increased or decreased starvation resistance, likely depending on whether triglycerides stored in lipid droplets can be mobilized. For example, flies with the Brummer lipase gene loss-of-function mutation are obese and starvation sensitive [23], while overexpression of *Lsd–2* (encoding a perilipin protein), leads to an obese and starvation-resistant phenotype, reminiscent of our flies with deficient midgut acidity [24]. Furthermore, increased weight or lipid content is not necessarily linked to decreased life span. The *methuselah* mutant fly is heavier, and starvation resistant, but also long-lived [25]. A recent study by Lee [26] also showed a positive (and almost linear) correlation between lipid content and life span in female flies fed diets having various protein:carbohydrate ratios. Nonetheless, excessive lipid storage may still increase the risk of mortality in insects [27].

Parallel change in life span and stress resistance (starvation resistance, in particular) has often been noted in *Drosophila* studies [28]. For instance, flies with loss of the Apolipoprotein D (ApoD) homolog exhibit reduced life span and lowered starvation resistance [29], while overexpression of the ApoD homolog leads to life span extension and starvation resistance (without alteration of lipid content) [30]. Other examples include the *methuselah* mutant and female flies with loss of chico, both being long-lived and starvation resistant [25, 31]. Furthermore, starvation resistance has been successfully used as a screening strategy for identification of longevity genes [32]. However, our study makes the case that the two can be decoupled, and that caution should be exercised in using one to infer the other.

An aspect of digestion that is conserved between flies and mammals is the role of an acidified gut compartment. Even yeast, a unicellular organism in which the vacuoles serve as digestive organelles could be seen as conserving this strategy. Interestingly, *vha* is also responsible for vacuolar acidification, and suppression of vacuolar acidity in yeast affects nutrient signaling, leading to shortened replicative life span [33]. Together with our findings, these observations suggest the intriguing possibility that the relationship between digestive organ/organelle acidity and life span may be evolutionarily conserved.

In addition to supporting the robustness of findings observed in genetic models, another benefit of using pharmacological models in our study derives from the fact that suppression of midgut acidity is limited to adult fly life. This helps to exclude the possibility that suppression of larval midgut acidity could contribute to some of the phenotypes observed in the genetic models, although a previous study has suggested that larval midgut acidity may be dispensable [34]. Importantly, pharmacological models have direct clinical relevance since acid suppression is a commonly used treatment for both peptic ulcer disease and gastroesophageal reflux disease in human patients. Indeed, long-term treatment with PPI was shown to be associated with altered intestinal microflora [35, 36] and with undesirable weight gain [4], the latter being consistent with our finding in flies. These previous reports, along with our findings, raise serious questions, and call for careful studies of the potential risks that may be associated with acid suppression therapy in humans.

In conclusion, our study adds to the organismal-level understanding of the inter-relationships between gut homeostasis, metabolism, and aging, in which gut acidity plays a role. Flies and mice with deficient gut acidification recapitulate features of metabolic syndrome, and thus could be candidates for disease modeling. Further study is needed to elucidate precisely how gut acidity acts to modulate various aspects of gut homeostasis and metabolism, and it would be of great interest to test whether preservation of gut acidity, if feasible, can extend life span.
